# Spatial Geometries of Self-Assembled Chitohexaose Monolayers Regulate Myoblast Fusion

**DOI:** 10.3390/ijms17050686

**Published:** 2016-05-06

**Authors:** Pornthida Poosala, Hirofumi Ichinose, Takuya Kitaoka

**Affiliations:** 1 Graduate School of Bioresource and Bioenvironmental Sciences, Kyushu University, 6-10-1, Hakozaki, Higashi-ku, Fukuoka 812-8581, Japan; pin_acocyte@agr.kyushu-u.ac.jp; 2 Faculty of Agriculture, Kyushu University, 6-10-1, Hakozaki, Higashi-ku, Fukuoka 812-8581, Japan; ichinose@agr.kyushu-u.ac.jp

**Keywords:** cell fusion, chitohexaose, glucose uptake, GLUT4, micropattern, myoblast, myosin heavy chain, skeletal muscle

## Abstract

Myoblast fusion into functionally-distinct myotubes to form *in vitro* skeletal muscle constructs under differentiation serum-free conditions still remains a challenge. Herein, we report that our microtopographical carbohydrate substrates composed of bioactive hexa-*N*-acetyl-d-glucosamine (GlcNAc6) modulated the efficiency of myoblast fusion without requiring horse serum or any differentiation medium during cell culture. Promotion of the differentiation of dissociated mononucleated skeletal myoblasts (C2C12; a mouse myoblast cell line) into robust myotubes was found only on GlcNAc6 micropatterns, whereas the myoblasts on control, non-patterned GlcNAc6 substrates or GlcNAc6-free patterns exhibited an undifferentiated form. We also examined the possible role of GlcNAc6 micropatterns with various widths in the behavior of C2C12 cells in early and late stages of myogenesis through mRNA *expression* of *myosin heavy chain* (*MyHC*) isoforms. The spontaneous contraction of myotubes was investigated via the regulation of glucose transporter type 4 (GLUT4), which is involved in stimulating glucose uptake during cellular contraction. Narrow patterns demonstrated enhanced glucose uptake rate and generated a fast-twitch muscle fiber type, whereas the slow-twitch muscle fiber type was dominant on wider patterns. Our findings indicated that GlcNAc6-mediated integrin interactions are responsible for guiding myoblast fusion forward along with myotube formation.

## 1. Introduction

Myoblast fusion is an indispensable step in the cell maturation process during the development and regeneration of adult skeletal muscular cells. At the cellular level, the fusion process of myoblasts is mainly involved in two characteristic stages: the phenotypic conversion and the subsequent transformation into multinucleated myotubes. Initially, the alignment of myoblasts was identified as critical for myoblast fusion via migration, recognition and rearrangement of the actin cytoskeleton at contact sites between two myogenic cells and the material surfaces. Then, membrane fusion occurs, leading to myoblast-myoblast fusion, which promotes the formation of nascent myotubes. Finally, growth of muscle fibers is accomplished by nuclei acceleration and the formation of more mature myotubes [1,2,3,4].

The differentiation of skeletal myoblasts (C2C12; a mouse myoblast cell line) and myotube maturation *in vitro* requires switching from growth medium to differentiation medium, to decrease growth factors and slow cell proliferation, resulting in withdrawal from the cell cycle. Consequently, cell differentiation is promoted, and muscle-specific gene expression is initiated [5,6]. However, serum-free culture has been required for tissue engineering, to avoid immune rejection for *in vitro* applications. Thus, it has been reported that cells were treated under either serum-free or differentiation serum-free conditions [7,8,9]. Stevenson *et al.* [10] have shown that the cessation of feeding of myotubes in cell culture by starvation without media and serum replenishment resulted in the formation of significantly thinner myotubes and subsequent reduction in diameter, leading to rapid atrophy compared to control cells. Starvation atrophy in myotubes is presumed to be involved in the generation of reactive oxygen species, including changes in the transcription profiles [10]. However, inhibition of apoptosis in response to the regulation of stress fibers seems likely to be related [7]. Therefore, achieving direct stimulation of myoblast differentiation and fusion on the substrate in culture with growth serum medium without any addition of differentiation factors would be a promising progress in the development of functional muscles for *in vitro* applications. In addition, many studies have revealed that signaling pathways regulate various transcription factors and have provided significant information on the molecular mechanisms of cell fusion by the cooperative crosstalk of the core signal transduction machinery of each pathway [11,12,13,14]. The understanding of these pathways has deepened through biomolecular interactions, occurring at cell-surface receptors via intracellular domain-associated signaling or adaptor proteins inside the cells. Numerous types of natural bioactive molecules, which are immobilized on cell culture scaffolds, can immensely affect myoblast functions, particularly encouraging myotube formation and contractibility [15,16,17,18].

*In vitro*, the geometrically-confined topography at the micro- and nano-scale features influences the enhancement of myoblast fusion into myotubes by affecting the reorganization of the cytoskeleton and focal adhesion kinase (FAK) [19,20,21]. The physicochemical properties of cell culture scaffold materials have been implicated to assist in mimicking the three-dimensional microenvironment conditions and represented the attainable goals of controlling cell behaviors and cell responses. In our previous studies, we have determined the cellular activities and responsiveness on self-assembled monolayers (SAMs) of carbohydrate oligomers and their derivatives, which were immobilized on gold surfaces [22,23,24]. Interestingly, our findings have demonstrated that carbohydrate clustering holds great potential for controlling biophysical aspects of the microenvironment, including transducing extracellular signals to intracellular signaling cascades through diverse carbohydrate-mediated specific recognition at cell surface receptors [25]. In this study, we provide new insight into direct stimulation of myoblast cell fusion by incorporating chitohexaose (hexa-*N*-acetyl-d-glucosamine (GlcNAc6)) oligomers in microtopographical cues, to promote the differentiation of myoblasts under culture conditions without the addition of horse serum or any other differentiation media. C2C12 cells were preferentially aligned along the chitohexaose patterns (GlcNAc6-SAMs) and demonstrated myotube-like characteristics. The GlcNAc6-SAMs, containing six *N*-acetyl-d-glucosamine (GlcNAc) residues, may be attractive for practical engineering of customized muscle-like tissue architectures. We anticipated that the parallel alignment of myoblasts would consequently facilitate cell fusion by promoting the contractile activity of cells through contraction-stimulated glucose uptake and regulation of glucose transporter 4 (GLUT4), a major glucose transporter, which has been identified and is predominantly expressed in C2C12 myoblasts [26]. Additionally, we revealed that GlcNAc6-SAM patterns enhanced the relative mRNA expression level of myosin heavy chain isoforms (*MyHCs*). *MyHCs* are characterized as regulators that lead to a major change in cell membrane transformation during the late stage of myogenic differentiation, contributing to variations in contractile dynamic characteristics in myoblasts [27,28,29]. Thus, in this study, we demonstrated that our established carbohydrate-functionalized surface geometries enable the formation of robust myotubes from myoblasts without adding any culture differentiation medium. These findings are a crucial step toward tissue engineering and may have a great therapeutic potential in the future.

## 2. Results

### 2.1. Effects of Topographical Features and Chitooligomers on Myotube Formation

**Figure 1:**
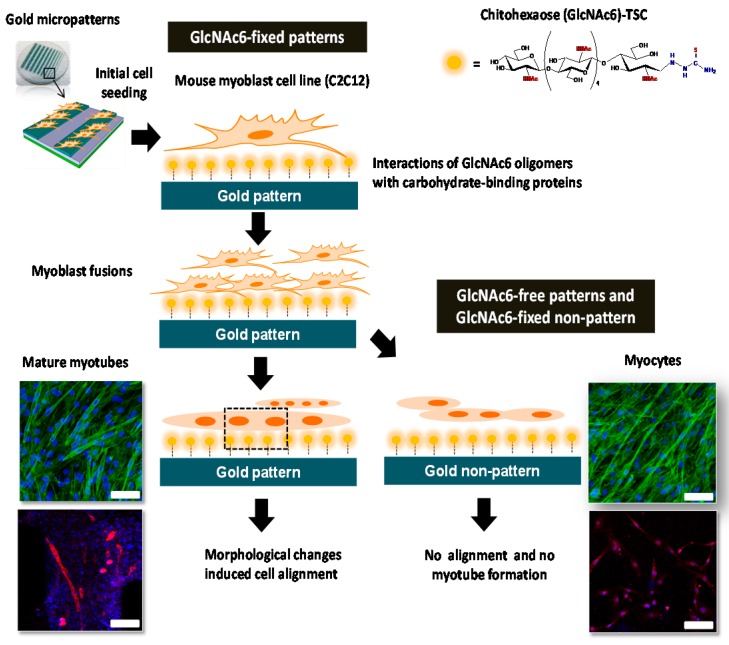

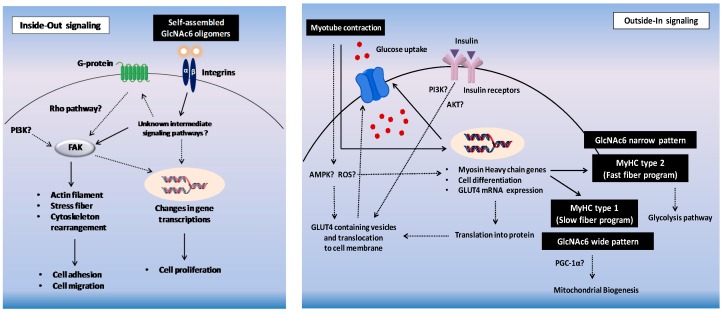
Schematic illustration of differentiation behavior and myoblast fusion on microscale topographical patterns of hexa-*N*-acetyl-d-glucosamine (GlcNAc6)-self-assembled monolayers (SAMs) and GlcNAc6-free substrates, directing myotube formation and the possible cellular signaling machinery involved in gene regulation and contraction-stimulated glucose uptake through a specific GlcNAc6–receptor interaction on cell surfaces, which denoted in a dashed black box. The main signaling pathways for possible regulation through GlcNAc6 oligomers interacting with glyco-receptor proteins in myoblasts (thick arrows) and the convergence of unknown signaling pathways that induce myoblast fusion (dashed arrows) are depicted. Scale bars represent 200 µm.

**Figure 2:**
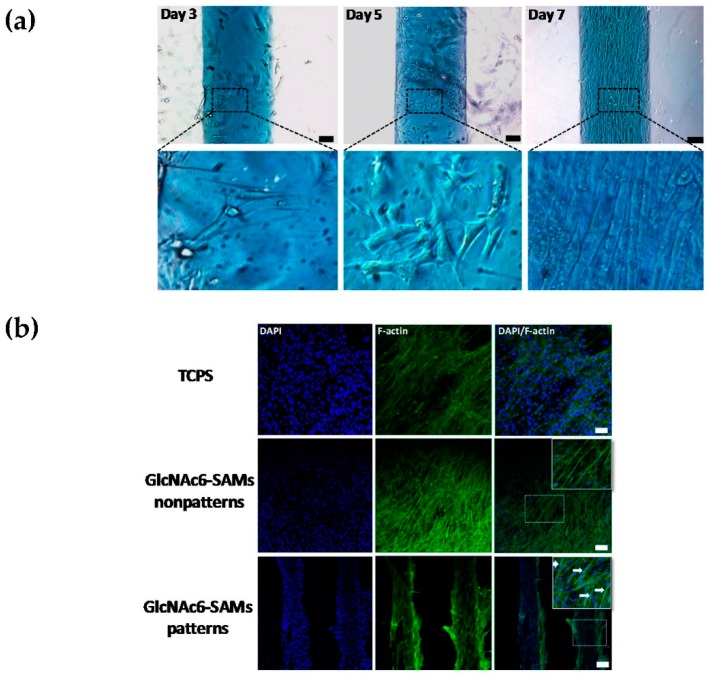

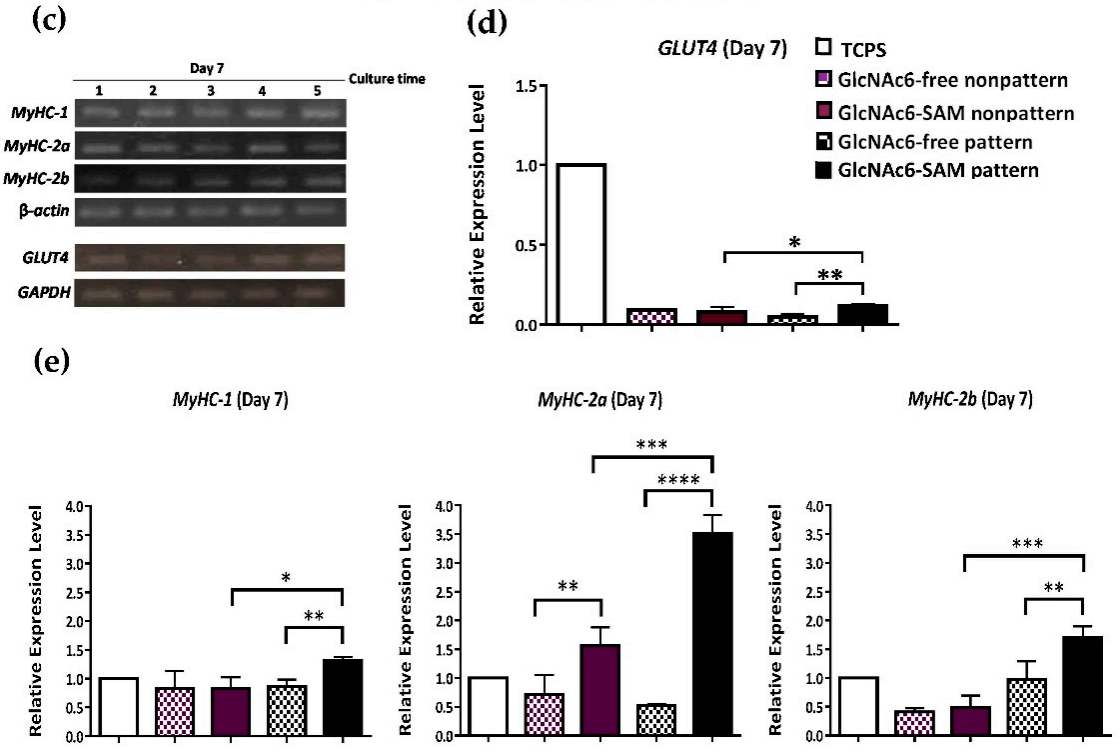
Effects of micropatterns and non-patterns with or without GlcNAc6-SAMs on differentiated C2C12 cells during myotube development. (**a**) Myoblast differentiation proceeds in stages from Days 3–7 under culture medium without switching to differentiation medium on the GlcNAc6-SAM pattern (1000 µm); (**b**) Confocal images of differentiated myoblasts on the GlcNAc6-SAM pattern (500 µm). Actin filaments were stained green, and nuclei were visualized with DAPI (blue) after seven days of culture. Myotubes are labeled with white arrows. Scale bars represent 200 µm (**c**–**e**). mRNA expression levels of *GLUT4* and three isoforms of myosin heavy chains (*MyHCs*) in myoblasts after seven days of culture. The expression level was normalized to *GAPDH* and β*-actin* for *GLUT4* and *MyHCs*, respectively. Representative polymerase chain reaction (PCR) products of target genes were determined on 2% agarose gels by ethidium bromide staining. Lane 1: tissue culture polystyrene (TCPS); Lane 2: GlcNAc6-free non-pattern; Lane 3: GlcNAc6-SAM non-pattern; Lane 4: GlcNAc6-free pattern (500 µm); and Lane 5: GlcNAc6-SAM pattern (500 µm). Asterisks signify a significant difference from the appropriate control value. Values are the mean ± SEM, *n* = 9 per each sample; * *p* < 0.05, ** *p* < 0.01, *** *p* < 0.001 and **** *p* < 0.0001.

We successfully fabricated a synergistic combination of topographical characteristics and GlcNAc6 oligomers self-assembled in two different geometries, as depicted in Figure 1, according to our previous report [25]. Here, C2C12 cells were cultured on GlcNAc6-SAMs or GlcNAc6-free substrate, to confirm our hypothesis that immobilized GlcNAc6 oligomers promote myoblast fusion and to demonstrate direct myotube formation in a culture system without any replenishment of differentiation medium. We found that the myoblasts started to fuse together on GlcNAc6-SAM patterns after five days of culture (Figure 2a) and subsequently promoted unidirectional cell orientation, as well as actin alignments after seven days of culture (Figure 2b). In contrast, myocytes and undifferentiated C2C12 cells were observed on GlcNAc6-free patterns, the GlcNAc6-SAM non-pattern and control tissue culture polystyrene (TCPS) substrates. This result suggests that physical structures of the underlying GlcNAc6-SAM substrates can influence the clustering of integrins and other adhesion molecules, which in turn activate singling pathways that ultimately govern cell behavior. It is possible that such an interaction occurs at the interface of GlcNAc6 oligomers and integrin-based adhesion complexes, which are a prerequisite for myotube formation.

To gain more insights into the influence of topography-mediated myoblast differentiation on myotube functions, we first evaluated the mRNA expression levels of *GLUT4* and MyHC isoforms, which have a significant role in the indirect physiochemical mechanism of the spontaneous contractions of differentiated myoblasts. At this stage, experiments were conducted at Day 7 after the initial cell seeding, according to the late stage differentiation of C2C12 cells. GlcNAc6-SAM-fixed patterns demonstrated a statistically-significant higher expression of the GLUT4 and MyHC genes, compared to that on the GlcNAc6-SAM non-pattern or GlcNAc6-free patterns (Figure 2c–e). This indicated that primary myotubes preferentially form themselves on GlcNAc6-SAM substrates. These results are consistent with our prior finding that clustered carbohydrate patterns regulated more effectively myoblast behavior than carbohydrate-free substrates [25]. Topographical features may effectively direct myotube maturation; thus, we examined whether micropatterned GlcNAc6-SAM substrates play a vital role in transcriptional regulation (up- or down-regulation) of the activation of mechanotransduction signaling, which is involved in subsequent myoblast fusion. The following experiments were undertaken on GlcNAc6-SAMs, which were designed to have the exact dimensions of micropatterned widths of 200, 500 and 1000 µm.

### 2.2. Hexa-N-acetyl-d-glucosamine (GlcNAc6)-self-assembled Monolayers (SAMs) Regulate Glucose Transporter Type 4 (GLUT4) mRNA Expression

**Figure 3:**
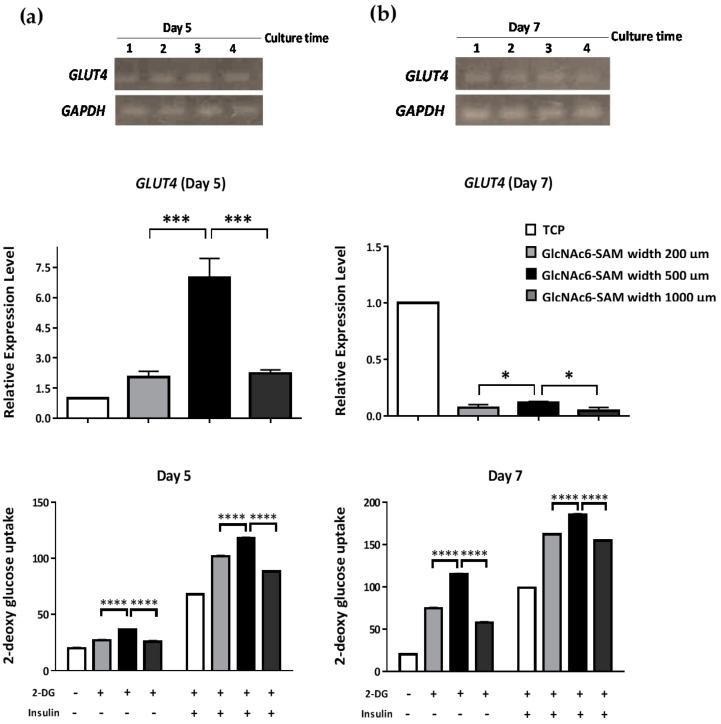
Micropatterned GlcNAc6-SAMs induce *GLUT4* mRNA expression in differentiated C2C12 cells through contraction-dependent glucose uptake. Myoblasts were stimulated with (+) or without (−) 1 µM insulin, and glucose uptake rates were measured. Data were obtained after (**a**) five days and (**b**) seven days. Results are presented as the mean ± standard error of the mean (SEM) from triplicate measurements and are representative of three independent experiments. Lane 1: tissue culture polystyrene (TCPS); Lane 2: GlcNAc6-SAM pattern (200 µm); Lane 3: GlcNAc6-SAM pattern (500 µm); Lane 4: GlcNAc6-SAM pattern (1000 µm). Values were analyzed by the *t*-test, * *p* < 0.05, *** *p* < 0.001 and **** *p* < 0.0001.

GLUT4 expression is highly regulated in muscle cells. The translocation of GLUT4 to the plasma membrane surface is activated independently by an insulin-stimulated signaling pathway or cell contraction [30,31]. In this study, we investigated the mRNA expression level of *GLUT4* at five and seven days of culture on designated GlcNAc6-SAM patterns with different widths. *GLUT4* overexpression was detected at Day 5 on the GlcNAc6-SAM pattern (500 µm; Figure 3a). Furthermore, the expression levels on patterns of 200 and 1000 µm were slightly higher than those on the control substrates. Nevertheless, *GLUT4* expression was substantially reduced at Day 7, and the amount of mRNA was hardly detected. This implied that the mRNA of *GLUT4* is translated to produce GLUT4 protein, which in turn activates various protein cascades, including translocation of the GLUT4 transporter to the plasma membrane and response to glucose influx. We confirmed this through glucose uptake by C2C12 cells, as the level of 2-deoxy-d-glucose uptake (2-DG) markedly increased, in a time-dependent manner (Figure 3a,b). Besides, we found that the role of glucose uptake into cells depended on the width of the patterns. Particularly, the GlcNAc6-SAM pattern with a 500-µm width showed significantly higher 2-DG uptake than that of other substrates and the control.

Furthermore, we compared the 2-DG uptake in the presence or absence of insulin in myoblasts on GlcNAc6-SAM patterns. Insulin stimulates glucose uptake in muscle cells through activation of the translocation of GLUT4-containing vesicles from the cytosol to the plasma membrane through a protein phosphorylation cascade [32,33]. Our results showed that the narrow micropatterns improved the glucose uptake (Figure 3a,b). This indicated that GlcNAc6-SAM patterns directly stimulated glucose transport through an unknown mechanism, which is different from insulin-independent pathways during cell-induced contraction. Taken together, these findings suggest that our established GlcNAc6-SAM patterns have a strong influence on inducing myoblast differentiation into myotubes without using any differentiation medium.

### 2.3. Early and Late Stages of Myoblast Fusion on GlcNAc6-SAM Patterns

Myoblast fusion in skeletal muscle is an essential early step indispensable for the generation of multinucleated myofibers during *in vivo* muscle development. *MyHC*, a motor protein of muscle thick filaments that performs in the generation of mechanical force in skeletal muscle contraction, has fundamental roles in the dynamic regulation of myoblast fusion [9,34]. The biochemical properties of muscle fibers are involved in the speed of contraction and ATPase activity, in which *MyHC* type 1 (*MyHC-1*) has been shown to be responsible for slow contraction, whereas *MyHC-2* is responsible for fast contraction [35,36,37]. In this study, we examined whether different geometries of GlcNAc6-SAM micropatterns can directly influence myoblast differentiation and its spontaneous contraction in a differentiation serum-free medium.

**Figure 4:**
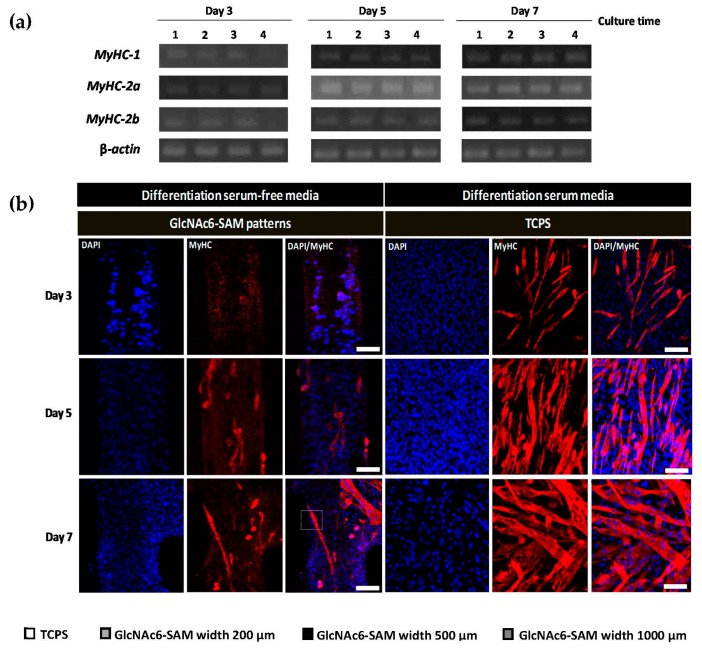

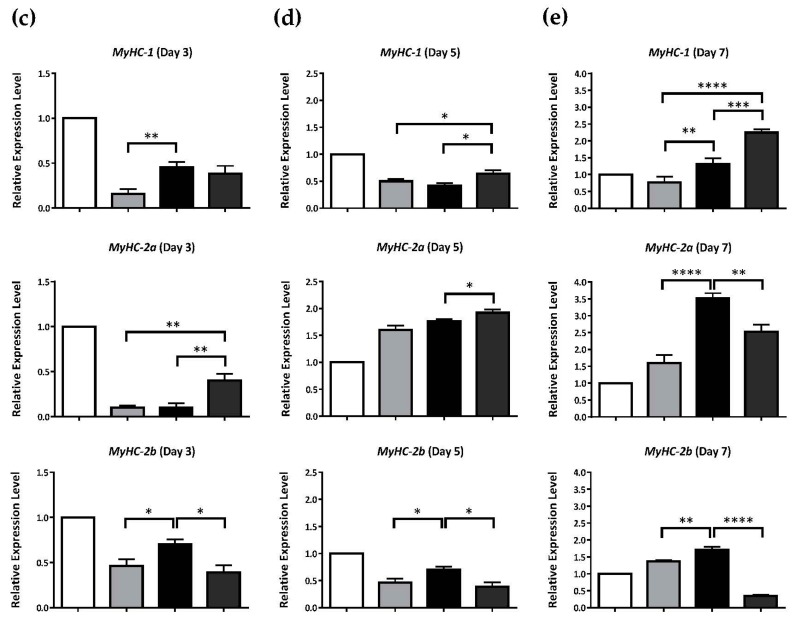
Effects of micropatterned GlcNAc6-SAMs on myosin heavy chain (MyHC) expression in differentiated C2C12 cells at different culture time points in a differentiation serum-free medium. (**a**) Representative quantitative real-time PCR products of *MyHC*s. The mRNA levels were normalized to β*-actin*. Lane 1: tissue culture polystyrene (TCPS); Lane 2: GlcNAc6-SAM pattern (200 µm); Lane 3: GlcNAc6-SAM pattern (500 µm); Lane 4: GlcNAc6-SAM pattern (1000 µm); (**b**) Immunocytochemical staining of MyHCs on narrow patterns and control TCPS substrate in the absence and presence of differentiation serum media, respectively. Myoblast fusion and myotube formation were found on GlcNAc6-SAM patterns after Day 5 and 7 of culture, respectively. A close-up image of multinucleated myotubes is shown in the inset panel. At Day 7, myotubes formed on the GlcNAc6-SAM patterns under differentiation serum-free conditions demonstrated long and thin morphology when compared to those on the TCPS substrate under differentiation serum-containing conditions, which promoted the formation of thicker myotubes with numerous branched structures. Shown are confocal images of differentiated cells stained with anti-MyHC antibody to monitor myotube formation (red). DAPI was used to visualize nuclei (blue). Scale bars represent 200 µm; (**c**–**e**) Individual *MyHC* mRNA expression profiles on various geometries. Values are the mean ± SEM, *n* = 9 per each sample. * *p* < 0.05, ** *p* < 0.01, *** *p* < 0.001 and **** *p* < 0.0001.

This hypothesis was assessed by validation of the gene expression of *MyHCs*, of which three individual isoforms have been identified as *MyHC-1*, *MyHC-2a* and *MyHC-2b*. The different *MyHC* isoforms have very different individual characteristics for defining specific muscle properties in mammalian adult skeletal muscle cells. Comparison of the mRNA expression levels revealed that at three days after cell seeding, there was only a trace amount of *MyHCs* detected (Figure 4a,c). Furthermore, low intensity staining of cells was visualized on substrates (Figure 4b), suggesting that nearly no myoblast fusion occurred.

However, the expression levels of *MyHc-2a* on all GlcNAc6-SAM patterns increased between Day 3 and 5 of cell culture by eight-fold on GlcNAc6-SAM with narrow patterns and by five-fold on the wide pattern (Figure 4c,d). Conversely, we observed no changes in the mRNA expression level of MyHC-2b. However, there was a slight increase in MyHC-1 expression on the narrow pattern (200 µm) and wide pattern (1000 µm). These results suggest that cultured myoblasts on GlcNAc6-SAM patterns underwent differentiation with complicated changes in morphology and were subsequently able to drastically transform. Interestingly, the initial stage of myoblast-induced cell fusion seems likely to depend on *MyHc-2a* gene expression.

The expression level of *MyHC-1* on the wider pattern (1000 µm) was higher than that on the narrow patterns between Day 5 and 7 of cell culture; however, the level of *MyHC-2b* expression did not significantly change during myoblast development (Figure 4c–e). Conversely, the narrow patterns showed higher expression of *MyHC-2b* and consistently altered in the expression levels of both *MyHC* type 2 isoforms (Figure 4d,e). To compare the morphological changes of myotubes in the late stage of muscle differentiation, herein, myoblasts were cultured in differentiation serum-free and differentiation serum-containing media. Immunostaining at Day 7 confirmed that the myoblasts were completely transformed into myotubes by fusion on GlcNAc6-SAM patterns under differentiation serum-free conditions, showing the long and thin appearance of myotubes. By contrast, control TCPS substrate promoted thicker myotubes with numerous branched structures when compared to those of GlcNAc6-SAM patterns (Figure 4b). This indicated that more mature myotubes were found on the control substrate, due to the effect of differentiation media. However, these results revealed that GlcNAc6 oligomer-fixed geometries enhanced the fusion efficiency of myoblasts and greatly influenced the regulation of myotube formation without requiring any differentiation medium during cell culture, eventually leading to muscle contraction.

## 3. Discussion

During *in vitro* differentiation, the biophysical cues of topographical features alter and regulate a variety of cellular processes. Many researches have given important evidence regarding the mechanisms and biomolecules that mediate myoblast fusion in skeletal muscle [38,39,40]. However, a complete understanding of the precise mechanisms that lead to the activation and recruitment, governing the fusion process, still remains enigmatic. In this study, we highlighted the synergistic effect of combining microscale topographical patterns and surface chemical functionality using chitooligomers with bioactive motifs on the development of structured myoblast cultures under differentiation medium-free conditions. Our data clearly demonstrated that carbohydrate-functionalized micropatterns induced transcriptional activation of the *MyHC* and *GLUT4* genes, which results in the regulation of numerous downstream signaling pathways related to myoblast fusion and myotube contractions. In this work, we believe that Rho-family GTPases play critical roles in a major signaling pathway during myogenesis induction and myoblast fusion. This is because the Rho GTPases make an essential contribution to regulating cell adhesion, migration, cell proliferation and differentiation, particularly in skeletal myogenesis [41,42,43,44]. During a multistep process of myoblast fusion, actin cytoskeleton dynamics regulate the organization of the actin filaments, which subsequently control myoblast alignment and elongation. Our findings showed that GlcNAc6 oligomers play a predominant role in the transition of primitive myoblasts into myotubes by concomitant drastic reorganization of actin filaments and the contractile capabilities of cells [45,46,47,48]. We anticipated the direct binding of GlcNAc6 to integrin receptors on the cell surfaces to be an important factor in the modulation of mechanotransduction, which presumably occurs to trigger downstream signal pathways that subsequently act as the driving force for myoblasts fusion, including the influence of actin polymerization, leading to changes in cell phenotype and cell behaviors, as well as the cell fate decision.

During myoblast differentiation, a significant difference in the morphological changes of cells on GlcNAc6-SAM patterns was observed after 3–7 days of initial seeding. The initial formation of nascent myotubes, which is referred to as the second stage of myoblast fusion, was found at Day 5. Subsequently, myoblasts were fused with nascent myotubes into multinucleated myotubes along with high spatial regulation of aligned myoblasts at Day 7. The long-range self-organization of myoblasts is required to organize muscle architecture aligned with neighboring tissues through contact guidance in order to exhibit proper muscle function. The unidirectional alignments occur only in differentiating myoblasts, as indicated by the phase contrast images and fluorescence immunostaining of myosin. The effects of physical changes on the direction of myoblast alignment have been associated with actin filaments through activation of integrin-mediated signaling pathways [25]. This indicated that the GlcNAc6 oligomers fixed on the substrates performed to activate the signaling pathways during the first phase of myoblasts fusion.

We determined the effects of GlcNAc6-SAM patterns on the mRNA expression of *MyHC* isoforms. MyHCs are the main determinants of the contractile properties of adult mammalian skeletal muscles, which have been associated with the stage of myotube formation [49,50]. Myotube formation and maturation were subsequently achieved after seeding of seven days on GlcNAc6-SAM patterns. Our results revealed that the type of *MyHC* isoform expressed was predominantly dependent on the width of the GlcNAc6-SAM patterns. Narrow patterns tended to regulate *MyHC* type 2, whereas *MyHC* type 1 was profoundly expressed on the wide pattern. This implied that narrow patterns preferentially upregulated the expression of *MyHC* type 2 upon cell commitment and induced drastic downregulation of *MyHC* type 1 with time, due to the rapid significant increase in myoblast fusion, which later transformed into myotubes. This suggests that the onset of cell cycle withdrawal can be greatly accelerated by narrower patterns and demonstrated an early differentiation phase. Furthermore, the effects of narrow dimensions on end-to-end alignment of myoblasts are a synergistic factor for enhancing fusion and differentiation into myotubes, because myoblasts are polarized cells, and the decrease in the width of the topographical features increased the migration rate, resulting in enhanced recognition and adhesion of myoblasts prior to fusion [51]. However, it has been reported that myoblasts were not able to fuse on narrow substrates despite cells being highly aligned in one direction and exhibiting lateral contacts with neighboring cells [52]. Because of these discrepancies, we confirmed that GlcNAc6-SAMs play a key role in myoblast fusion through aligned assembly of myoblasts to promote the subsequent fusion and differentiation into multinucleated myotubes.

The presence of spontaneous contractile activity in cultured myotubes on GlcNAc6-SAM patterns was confirmed through *GLUT4* mRNA expression and glucose uptake. Interestingly, elevation in glucose uptake and reduction in *GLUT4* mRNA levels at Day 7 seem plausible with regard to changes in *MyHC* type 2 expression, which is possibly relevant to fast-twitch muscle fibers. We speculated that GlcNAc6 oligomers on patterns induced conformational changes in the cross-bridge action where myosin-binding sites on actin molecules result in enhanced myosin ATP concentration and contraction velocity. An increase in contraction speed is presumably responsible for releasing intracellular GLUT4 vesicles through depletion of *GLUT4* mRNA levels, leading to increased regulation of GLUT4 translocation and trafficking to the cell membrane to facilitate glucose transport [53,54,55]. This implies that metabolic feedback signals and mechanical stress-activated signals are sufficient to elicit the full contraction glucose transport response during cell contraction [56].

In addition, we found that the molecular mechanisms controlling muscle plasticity are highly dependent on narrow GlcNAc6-SAM patterns during the fusion process. Interestingly, the 500-µm narrow pattern manipulated myoblast fusion more effectively compared with the 200-µm pattern, as determined by the glucose uptake rate and the mRNA expression level of MyHCs. This indicated that the predominant factor in manipulating myoblast fusion on narrow patterns was driven by the GlcNAc6-mediated actomyosin contraction. Taken together, our findings showed that GlcNAc6-SAM patterns play a pivotal role by being an intermediate regulator in modulating myoblast fusion. However, further investigation is required, to identify the functions downstream of small Rho GTPases, which generally promote actin polymerization and drive the formation of actomyosin filament bundles, including the associated regulators that are involved in contraction. Metabolic markers, such as AMP-activated protein kinase, reactive oxygen species and mitochondrial biogenesis, also need to be investigated, to obtain a better understanding of the convergence of multiple signaling pathways that lead to the activation of the signaling mechanisms of myoblast fusion on geometrical carbohydrate micropatterns.

## 4. Materials and Methods

### 4.1. Materials

Pure α-chitin powder from crab shells (Katakura Chikkarin, Tokyo, Japan) was hydrolyzed to a mixture of chitooligomers and then purified by gel filtration separation (Cellufine GCL-25, JNC Corporation, Tokyo, Japan) to obtain chitohexaose. Thiosemicarbazide (TSC, Wako Pure Chemical Industries, Osaka, Japan) and sodium cyanoborohydride (NaBH_3_CN, Sigma-Aldrich, St. Louis, MO, USA) were used as anchoring and reducing agents, respectively. The immortalized mouse muscle myoblast cell line, C2C12 (ATCC-CRL1772™, ATCC, Manassas, VA, USA), was subjected to cell culture assays. C2C12 cells were grown under the standard conditions with Dulbecco’s Modified Eagle’s Medium (DMEM, Life Technologies, Tokyo, Japan) supplemented with 10% fetal bovine serum (FBS; Biowest, Nuaillé, France). Tissue culture polystyrene (TCPS) dishes and TCPS plates (24-well) were purchased from Sumitomo Bakelite Co., Ltd. Tokyo (Japan). Trypsin–ethylenediaminetetraacetic acid (Trypsin–EDTA; Invitrogen, Tokyo, Japan) solution, 0.4% trypan blue solution (Invitrogen), phosphate-buffered saline (PBS; Nissui Pharmaceutical, Tokyo, Japan), phalloidin-Alexa Fluor^®^ 488 conjugate (Lonza, Walkersville, MD, USA) and molecular biology grade Triton^®^ X-100 (EMD Biosciences, San Diego, CA, USA) were used for biological assays. The water used in this study was purified with a Milli-Q system (Sartorius Stedim Biotech, Bohemia, NY, USA). All chemicals were of reagent grade quality and used without further purification.

### 4.2. Synthesis and Fabrication of Carbohydrate-Functionalized Monolayers on Gold Micropatterns

Chitohexaose (GlcNAc6) was prepared by acid hydrolysis and subsequently confirmed by chemical characterization as reported in our previous study [25]. Two-dimensional flattened micropatterns were designed with various intrinsic widths (200, 500 and 1000 µm), using template masks (Microtech Laboratory, Kanagawa, Japan). The patterned substrates were immersed in 1.0 M aqueous solution of chitohexaose conjugated with thiosemicarbazide (GlcNAc6-TSC) prior to subsequent initiation of cell culture. The formation of carbohydrate self-assembled monolayers (GlcNAc6-SAMs) was reproducibly achieved on gold micropattern surfaces using site selective conjugation with TSC at their reducing end groups, subsequent to depositing on the micropatterns through thiol chemisorption.

### 4.3. Cell Culture Assay

Mouse-derived C2C12 myoblasts were cultured in complete media consisting of DMEM supplemented with 10% FBS. Substrates were sterilized with ultraviolet (UV) light prior to cell seeding, as previously reported [25]. Briefly, 5.0 × 10^4^ cells were seeded on control (TCPS) or micropatterned substrates in a 24-well plate. To induce cell differentiation on the TCPS substrate for the immunostaining experiment, cells were grown to confluence, and cell culture was shifted to DMEM supplemented with 2% horse serum (Thermo Fisher Scientific Inc., Waltham, MA, USA). Differentiation medium was changed every day during 6 days of cell culture. Samples were incubated for 3, 5 and 7 days at 37 °C in an atmosphere of 95% air and 5% CO_2_ for immunostaining and mRNA gene expression analysis. The numbers of viable cells were counted using an automated cell counter (TC 20TM, Bio-Rad Laboratories, Inc., Philadelphia, PA, USA) after treatment with a 0.4% trypan blue solution. Cell behaviors and morphological changes were observed with a phase-contrast microscope (Leica DMI 4000B microscope, Wetzlar, Germany).

### 4.4. Quantification of mRNA by Real-Time Quantitative Polymerase Chain Reaction (RT-PCR)

Extraction of total RNA was performed using ISOGEN (Nippon Gene Co., Ltd., Toyama, Japan) according to the manufacturer’s instructions. cDNA templates were synthesized from 20 ng of total RNA and were reverse transcribed using PrimeScriptase RTase (Takara Bio Inc., Shiga, Japan), followed by triplicate polymerase chain reaction (PCR) reactions in 10 µL of SYBR Green Master Mix (Applied Biosystems™, Life Technologies, New York, NY, USA), containing 10 µM of forward and reverse primers. The primer sequences for each gene (Table 1) were designed by the primer 3 plus software and the NCBI PubMed primer blast software. Amplification and quantification of mRNA were performed at 95 °C for 20 s, followed by 40 cycles of 3 s at 95 °C and 30 s at 60 °C. After amplification, a melting curve analysis was carried out to verify the specificity of amplification products. The relative expression levels of target genes were normalized by subtracting the corresponding *GAPDH* and β*-actin* threshold cycle (*C*_t_) values and using the ΔΔ*C*_t_ comparative method [57]. PCR products were separated on 2% agarose gels, stained with ethidium bromide and then photographed under UV light to confirm a single amplicon.

### 4.5. Glucose Uptake Assay

The glucose uptake assay was carried out through measurement of the transport of accumulated 2-deoxy-d-glucose-6-phosphate (2-DG6P), which is proportional to 2-DG (or glucose) uptake by cells. C2C12 cells were cultured in 24-well plates for 5 or 7 days. After a 12-h serum starvation in DMEM, differentiated C2C12 cells were thoroughly rinsed three times with PBS, followed by incubation with Krebs-Ringer-Phosphate-HEPES (KRPH) buffer containing 2% bovine serum albumin (BSA) (KRPH: 20 mM Hepes, 5 mM KH_2_PO_4_, 1 mM MgSO_4_, 1 mM CaCl_2_, 136 mM NaCl, 4.7 mM KCl, pH 7.4) for 40 min. Then, the cells were stimulated with or without 1 µM insulin for 20 min in the presence of 10 mM 2-DG. The amount of glucose in the lysate was quantified using the Glucose-Uptake assay kit (ab136955, Abcam Inc., Cambridge, UK), and the absorbance values of samples were measured at 412 nm on an EnSight Multimode Plate Reader (PerkinElmer Inc., Waltham, MA, USA).

### 4.6. F-Actin Staining

Actin filaments were visualized by fluorescence staining of F-actin, and their alignment was determined. Cultured cells were rinsed twice with pre-warmed PBS, fixed with 3.7% (*v*/*v*) formaldehyde for 10 min, and permeabilized with 0.1% Triton^®^ X-100 in PBS for 5 min. Fixed cells were pre-incubated with 1% BSA in PBS for 20 min, to avoid non-specific protein binding and to increase fluorescence intensity. Filamentous actin was stained with Alexa Fluor^®^ 488-conjugated phalloidin (1:100; Lonza) according to the manufacturer’s protocol. Subsequently, the nuclei were stained with 4’,6-diamidino-2-phenylindole dihydrochloride (DAPI, 1:1000 in PBS; Nissui Pharmaceutical, Tokyo, Japan). Images of stained sections were acquired using a confocal microscope (Nikon ECLIPSE TE2000-U, Nikon Corporation, Kanagawa, Japan).

### 4.7. Immunostaining of MyHC

After 3, 5 and 7 days of cell culture in either differentiation serum-free or differentiation serum-containing media, C2C12 cells were rinsed with cold PBS and pre-fixed with 4% (*v*/*v*) formaldehyde in the culture medium for 2 min. Then, the medium was replaced with 2% (*v*/*v*) formaldehyde and incubated for 20 min at room temperature (RT). After washing twice with PBS and wash buffer (0.1% BSA in PBS), blocking buffer (PBS/10% normal donkey serum (ab166643)/0.3% Triton^®^ X-100) was added to avoid non-specific staining for 45 min at RT. Cells were then immunostained for myosin by incubation with a mouse monoclonal anti-MyHC antibody (MF-20, diluted in the PBS, 1:100; R & D Systems, Inc., Minneapolis, MN, USA), followed by the secondary antibody, which was visualized with a donkey anti-mouse IgG (H + L) antibody conjugated with Alexa Fluor 594 (NL007, 1:200 dilution; R & D Systems, Inc.), at RT for 1 h. Subsequently, the nuclei were stained with DAPI (1:1000 in PBS; Nissui Pharmaceutical). Fluorescence images at different magnifications were acquired to visualize the myotubes using a confocal microscope (Nikkon ECLIPSE TE2000-U, Nikon Corporation).

### 4.8. Statistical Analysis

Bioassay data of individual samples were separately analyzed in triplicate using GraphPad Prism Version 6.0 (GraphPad Software, Inc., La Jolla, CA, USA). Data are expressed as the mean ± SEM. Statistical differences between two groups were evaluated using an independent *t*-test. Statistical significance is indicated by *p*-values of: * *p* < 0.05, ** *p* < 0.01, *** *p* < 0.001 and **** *p* < 0.0001.

## 5. Conclusions

The underlying spatial geometries consisting of GlcNAc6 oligomers stimulated myoblast fusion and spontaneous contraction through various transient activations of fusion-related target genes. These micropattern geometries of GlcNAc6-SAMs affected individual facets of myoblast behaviors, demonstrating an increased fusion of mononucleated myoblasts into myotubes and alignment towards a preferential direction, as well as high expression of genes involved in muscle contraction. These distinctive responses were predominantly associated with narrow patterns of GlcNAc6-SAMs. Based on our findings, we concluded that GlcNAc6 residues mediated fusion machinery during muscle development in a differentiation serum-free culture system and suggest the significances of spatial geometries and glyco-mediated interactions in producing aligned skeletal muscle for tissue engineering applications.

## Figures and Tables

**Table 1:** List of primers used for validation of gene expression using quantitative real-time RT-PCR.

Target Gene	Forward Sequencing Primer (5’ to 3’)	Reverse Sequencing Primer (5’ to 3’)	Product Length	Accession Number
*GLUT4*	GTAACTTCATTGTCGGCATGG	AGCTGAGATCTGGTCAAACG	155	NM_009204
*MyHC-1*	GTCCAAGTTCCGCAAGGT	CCACCTAAAGGGCTGTTG	205	NM_080728
*MyHC-2a*	TGACCTTGAGCTGACACTGG	CGGTGCCACAGGCAAACTG	194	NM_001039545.2
*MyHC-2b*	CGGTGCCACAGGCAAACTG	AGAAGCATCTCAATAAGCTCTGGTT	150	NM_010855.3
*GAPDH*	CCGTGTTCCTACCCCCAATG	AAGCCCAGCTCTCCCCATA	82	NM_008084.3
β*-actin*	GATTACTGCTCTGGCTCCTAG	GACTCATCGTACTCCTGCTTG	147	NM_007393.5
